# Composite “Crosslinked Polyvinyl Alcohol-Magnetite” as a Stimuli-Responsive Matrix for Optical Methods

**DOI:** 10.3390/molecules29122794

**Published:** 2024-06-12

**Authors:** Ivan S. Shchemelev, Alexander V. Ivanov, Nikolay B. Ferapontov

**Affiliations:** 1 Department of Chemistry, Lomonosov Moscow State University, 119991 Moscow, Russia or sandro-i@yandex.ru (A.V.I.); n.ferapontov@gmail.com (N.B.F.); 2 Kurnakov Institute of General and Inorganic Chemistry of the RAS, 119071 Moscow, Russia

**Keywords:** composite stimuli-responsive (sensitive) materials, polyvinyl alcohol, magnetite, optical micrometry, digital color measurement

## Abstract

The preparation and application of the composite material “crosslinked polyvinyl alcohol—magnetite” as a sensitive matrix for use in digital colorimetry and optical micrometry methods are discussed. The material was synthesized in the form of spherical granules (for micrometry) and thin films (for digital colorimetry). The obtained composites were characterized by the registration of magnetization curves. It was shown that the amount of grown Fe_3_O_4_ particles in the polymer gel is in linear dependence with the iron salt concentrations in the impregnating solutions. The composite granules were applied to determining monosaccharides using optical micrometry. The optimal pH value for the total amount of monosaccharides’ determination was 8.6. The study of the analytical response of composite granules and films performed with a low limit of detection (7.9 mmol/dm^3^) of both glucose and fructose and a possibility of the control of high alcohol contention in water media. The granules were used to determine the total carbohydrate content in samples of natural honey and syrups with high fructose contents, while the films were used to control the alcohol content in hand antiseptics. The results obtained are in good agreement with the data provided by the manufacturers.

## 1. Introduction

Recently, in analytical chemistry, there has been an interest in the use of composite materials consisting of chemically different components forming a certain spatial structure as sensitive matrices. Such materials’ properties are determined by their components’ properties separately and together. A special case of such materials includes composites from “polymer—metal” or “polymer—metal oxide” compositions. Using these matrices makes it possible to increase sensitivity and selectivity in determining analytes within real samples [1].

Composites, as well as nanostructured materials of the polymer–metal/metal oxide composition, in which the size of inorganic particles ranges from 1 to 100 nm, have recently begun to be used as stationary phases in chromatographic analysis methods. Thus, stationary phases based on organometallic frameworks [2] and gold nanoparticles [3] are used for the chromatographic separation of optical isomers. Composite sorbents for the solid-phase extraction of metal ions [4,5], pesticides and insecticides [6,7], pharmaceutical substances [8], and toxins [9,10] with higher selectivity to target components compared to traditional synthetic and natural polymers have been proposed. Composite matrices are used in various chemical sensors, the response of which can be both electrochemical [11,12] and optical [13,14]. For example, for holographic sensor films and photonic crystals [15,16,17], swelling causes shifts in their Bragg diffraction, and the effective refractive index of the film also changes.

“Crosslinked polymer—magnetite” composites are of particular interest. The Fe_3_O_4_ particles embedded in the polymer matrix impart magnetic properties to the materials, simplifying the separation of the sorbent from the mother liquor in the case of static sorption. In chemical analysis, they have been used as sorbents for the preliminary separation and concentration of detectable substances [18]. Sorbents have been developed for the extraction and subsequent determination of narcotic and medicinal substances [19,20], heavy metal ions [21,22], and nitrofuran metabolites [23]. The production of Fe_3_O_4_ particles does not require expensive reagents.

A promising area of application for materials with “hydrophilic polymer—magnetite” compositions is their use as sensitive elements in the optical micrometry method, where the analytical signal is a change in the degree of their swelling in solution depending on their composition. Thanks to the magnetite particles embedded in the granule, it is possible to significantly increase the contrast of the granule against the background, as well as reliably fixing it with a magnetic field in the measuring cell, which significantly increases the convenience and accuracy of the measurements. The granular composite material “crosslinked polyvinyl alcohol (PVA)—magnetite”, proposed as a sensor matrix for the analysis of electrolyte solutions [24], organic acids, and carbohydrates [25], was studied in the most detail in the optical micrometry method. At the same time, the introduction of magnetite does not improve the low sensitivity and selectivity of PVA granules while maintaining the main disadvantage of the unmodified polymer: its low sensitivity and selectivity. The sensitivity of cross-linked PVA to carbohydrates can be increased due to its impregnation with a solution of sodium tetraborate. This approach increases the sensitivity of the glucose determination in model solutions by 30 times [26]. Using the example of determining the composition of natural honey samples using optical micrometry, it was shown that for impregnated PVA, the relative standard deviation for determining the total glucose and fructose content did not exceed 6%, and the obtained mass fractions of carbohydrates are in good agreement with the results of iodometric titration. However, tetraborate impregnation has not yet been used for PVA-magnetite granules.

On the other hand, the sensory material “polyvinyl alcohol-magnetite” can also be obtained in the form of films. The use of composites based on hydrophilic polymers with regularly distributed submicron silver particles as holographic sensors is documented in the literature’s data [16,17]. Such sensors are relevant for conducting express out-of-laboratory studies. The analytical response of such sensors (the color change) can be recorded using specular reflection spectrometry, or by processing the digital images obtained with a digital camera or scanner [27,28]. The best results are obtained in the case of processing photographic images obtained in raw formats [29], since, in this case, it is possible to carry out measurements in a wider spectral range. Composite films of “polyvinyl alcohol-magnetite” should also change color during swelling or compression in the analyzed solution by changing the distance between adjacent Fe_3_O_4_ particles. The simplicity of manufacturing PVA-based sensor films, as well as the low cost of the reagents used for this purpose, makes them a promising material for mass use in non-laboratory analysis methods. Therefore, it is of significant interest to applicate such films as sensor arrays when registering an analytical signal using a digital colorimetric method.

The purpose of this work was to study the possibility of using a composite material based on cross-linked PVA with submicron magnetite particles for the analysis of real objects in two forms: in the form of composite granules impregnated with sodium tetraborate for the determination of carbohydrates in syrup samples using optical micrometry; and in the form of composite films as sensor elements for the determination of ethanol and isopropanol when measuring an analytical signal using digital colorimetry. In the past, such composite granules have not been impregnated with a borax solution for the determination of carbohydrates. On the one hand, since the embedding of Fe_3_O_4_ particles into spherical polymer granules considerably expands the possibilities of optical micrometry, such an investigation is a very important step for the study of the application of optical micrometry as an analytical method for the control of real samples’ compositions. On the other hand, a novelty of this study lies in its use of composite polymeric films as sensitive matrices. Such analytical responses can be measured using modern available household gadgets containing photo cameras (such as digital cameras, smartphones, etc.). Thus, the proposed approach is a useful tool for simple, express out-of-laboratory analyses without the difficult pre-treatment of samples.

## 2. Results

### 2.1. The Study of Properties of Composite Granules

#### 2.1.1. The Characteristics of Composite Granules

**Figure 1:**
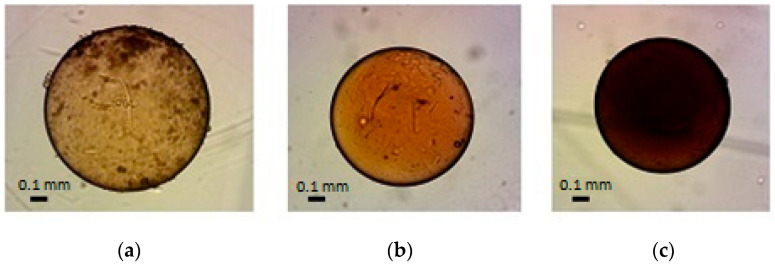
Images of the granules, captured during impregnation with Fe (III) and Fe (II) salt solutions with different concentrations: (**a**) 0.02 and 0.01 mol/dm^3^, (**b**) 0.05 and 0.025 mol/dm^3^, (**c**) 0.10 and 0.05 mol/dm^3^, respectively.

The applied technique for synthesizing the magnetite in the granules of cross-linked PVA is based on the one described in [25]. Magnetite particles were obtained via precipitation from iron (II) and iron (III) salts under the action of an ammonia vapor. In this case, the granules inside one batch may contain different amounts of magnetite. Mixing ammonia vapors with a fan makes it possible to distribute them evenly throughout the entire volume of the desiccator. The advantage of this deposition method is the narrower size distribution of the resulting particles due to the uniform filling of the reactor with the ammonia vapors [30]. When magnetite is deposited in a suspension of the PVA granules in an aqueous solution of iron salts, part of the magnetite is deposited on the surface of the granules, distorting their spherical shape and making it difficult to measure their size optically. To minimize this effect, the PVA granules were extracted from the mother liquor before magnetite deposition. The endurance time of the granules in the ammonia vapor was reduced to a day. Photographic images of the granules obtained in this way are shown in Figure 1.

The choice of iron salt concentrations for the impregnation of granules was determined by the necessity for sufficient contrast in photographs and also for the adequate transparency to select granules without defects, such as air bubbles, microcracks, or contour irregularities. Excessive concentrations of magnetite also reduce the osmotic stability of the granules: prolonged and repeated swelling and compression can cause solid magnetite particles to rupture the polymer granules.

**Figure 2:**
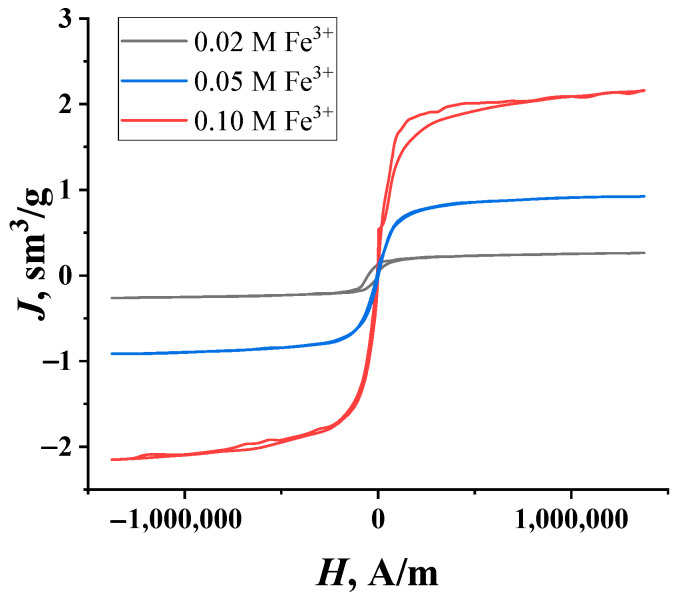
The magnetization curves of the composite granules of “PVA—magnetite” with different amounts of Fe_3_O_4_ particles.

The magnetic properties of the synthesized granules were tested through the registration of the magnetization curves shown in Figure 2. It is clearly seen that the hysteresis is almost absent in all curves. This result can be explained by the low coercive force connected with the very small mass fraction of Fe_3_O_4_ particles in the samples. Nevertheless, the linear proportion of iron salt concentrations in the impregnating solutions and the saturation magnetization that is typical for ferromagnetic materials persist.

The mass fraction of the magnetite in the granules was determined by comparing the saturation magnetization value with the magnetization curve of pure magnetite obtained separately. The high degree of purity of this standard (99.99%) was confirmed by X-ray phase analysis [31]. The higher the concentration of iron salts in the mother liquor, the higher the concentration of magnetite particles in the granules (Table 1), as confirmed by the difference in their color (Figure 1); this relationship is linear and is described by the equation:

*X* = 40.07*c* − 0.4 (1)

with a correlation coefficient above 0.99.

In a previous study [26], we demonstrated that the impregnation of the PVA gel with a sodium tetraborate solution increased the sensitivity of the determination of glucose and fructose as monosaccharides with hydroxyl and carbonyl groups in the structure 30-fold. Due to a selective ligand exchange process, that leads to the desorption of boron from PVA, occurred and caused a significant change in the degree of the gel’s swelling. However, all experiments were carried out exclusively on granules made of unmodified Fe_3_O_4_ polymer particles. The authors of the work [24] showed that the introduction of magnetite into granules of cross-linked PVA does not affect the degree of swelling of the polymer in electrolyte solutions. However, in more complicated systems, for example, using buffer solutions with complex compositions, chemical interactions of buffer solution components with Fe_3_O_4_ particles are possible, which may affect the degree of swelling of the sensitive polymer and increase the analytical signal.

In this work, we studied the effect of the amount of embedded magnetite on the degree of swelling of impregnated PVA in carbohydrate solutions. All measurements were carried out at pH values 6.8 and 8.6 for supporting solutions, corresponding to the maximum and minimum values of *V*/*V*_0_, respectively.

#### 2.1.2. The Effect of the Amount of Embedded Magnetite on the Concentration Dependencies of *V*/*V*_0_ in a Weakly Acidic Medium

At a pH of 6.8, the sensitivity of impregnated PVA to glucose and fructose is different: the change in the degree of swelling of PVA in the presence of fructose is noticeably higher, since the boron complex with fructose is more stable than its is with glucose [32]. At the same time, due to the ligand exchange process, transverse borate crosslinking is destroyed, leading to an increase in the volume of the PVA gel. Probably, this difference makes it possible to determine fructose and glucose separately if there is appropriate mathematical software.

**Figure 3:**
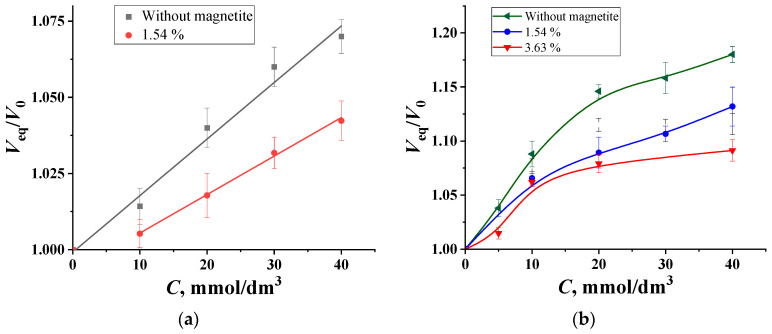
The concentration dependencies of the swelling degree of impregnated PVA granules with different amounts of magnetite in (**a**) glucose and (**b**) fructose solutions, with pH = 6.8, C_borax_ = 0.05 mol/dm^3^.

Figure 3 shows the dependencies of the relative volumes of granules of impregnated PVA from the concentrations of glucose and fructose in solutions at a pH of 6.8. In all these cases, the introduction of magnetite narrows the range of the detectable concentrations and reduces sensitivity. This can be explained by the fact that the volume of granules decreases by almost half when they are moved from water to the background, thereby doubling the concentration of magnetite particles within the granule volume. This leads, on the one hand, to a weakening of the electrostatic repulsion of boron-containing chelate ester groups located opposite each other and, on the other hand, to boron chemisorption on magnetite particles with the formation of groups (–O)_2_–B(–O–)_2_ on the surface. In this way, Fe_3_O_4_ particles form an additional binding bridge between polymer chains. This can also explain the decrease in the linearity of the concentration dependencies of the analytical signal for fructose solutions. Thus, due to the strong influence of magnetite, as well as the narrowness of the range of detectable contents, granules with magnetite should not be used for analytical purposes at a pH value of 6.8.

#### 2.1.3. The Effect of the Amount of Embedded Magnetite on the Concentration Dependences of *V*/*V*_0_ in a Basic Medium

**Figure 4:**
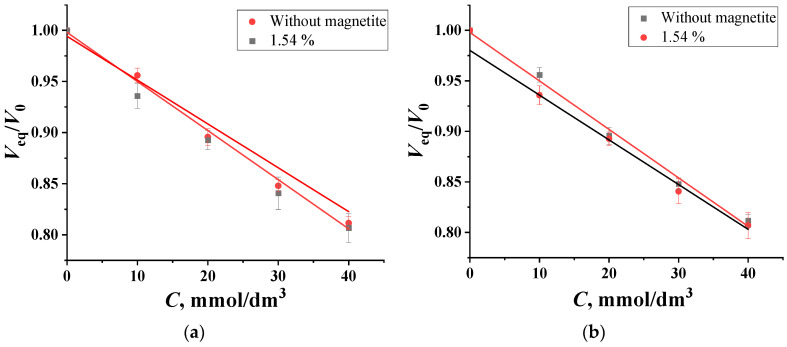
The concentration dependencies of the swelling degree of impregnated PVA granules with different amounts of magnetite in (**a**) glucose and (**b**) fructose solutions, with pH = 8.6, C_borax_ = 0.05 mol/dm^3^.

- At a pH value of 8.6, which promotes the formation of essential chelates of “PVA—boron” in a 1:1 composition, glucose and fructose are statistically indistinguishable from each other in terms of the swelling of the PVA. In this range, it is possible to determine the total content of the monosaccharides in natural honey samples using optical micrometry. The detection limit for glucose and fructose is 7.9 mmol/dm^3^ [26]. Sucrose, a disaccharide, does not provide an analytical response under these conditions because of the absence of *cis*-diol fragments in pyranose cycles. As can be seen from the dependence of the degree of swelling of granules on the concentration of carbohydrates shown in Figure 4, magnetite does not reduce the sensitivity of the polymer. In this case, when the granules are transferred from water to the supporting solution, their volume changes slightly, meaning that the concentration of magnetite particles also changes slightly. Additionally, under these conditions, the proportion of chelates in a 1:1 composition decreases significantly when carbohydrates enter the solution. Consequently, the charge of the polar groups of the impregnated polymer is not shielded by magnetite particles. The detection limits of glucose and fructose, respectively, do not change.

The statistical processing of the obtained dependencies showed that, as with unmodified PVA granules, glucose and fructose are statistically indistinguishable from each other. This makes it possible to use composite granules in the pH range of 8.5–10.5 for the total determination of glucose and fructose.

From the perspective of mechanical stability and the practical use of composite granules, the optimal mass fraction of magnetite in dried granules is 1.54%. Granules with this amount of magnetite content are dark enough to be detected with the naked eye, while still being transparent enough to detect defects such as air bubbles, irregularities, and cracks that can cause measurement distortion. This transparency allows for the pre-selection and exclusion of defective granules, leading to the improved reproducibility of the analytical response. As a result, the relative standard deviation of the *V*/*V*_0_ value is reduced from 2 to 1%.

#### 2.1.4. Analysis of Samples of Natural Syrups

- Based on the data obtained, it can be concluded that impregnated composite granules with a “cross–linked PVA—magnetite” composition are suitable for determining the total glucose and fructose contents in foods that do not contain other monosaccharides (for example, galactose). To confirm this, PVA granules with a magnetite content of 1.54% were used for the analysis of date syrup (Al Barakah Dates ©) and blue agave syrup (Agaven ©) that are used for diabetic nutrition and contain mainly fructose. Table 2 provides a comparison of the data on the total carbohydrate contents declared by the manufacturer and the values found using optical micrometry.

- As in the case analyzing samples of natural honey, the results obtained are in good agreement with the manufacturer’s data. With a more careful selection of granules (excluding defective ones), the standard deviation is reduced to 3 mass. %. Thus, using the composite granules based on PVA impregnated with sodium tetraborate, it is possible to analyze carbohydrate-containing products for the detection of counterfeits.

### 2.2. The Study of Composite Films’ Properties 

#### 2.2.1. Characteristics of Composite Films

Composites with different magnetite contents were manufactured as sensor arrays for photometric measurements. The raw material for their production was PVA grade 18/11, since the properties of the crosslinked polymer based on it had previously been well studied. At the same time, the average thickness of the films in one batch was 0.97 mm, which is very convenient for their further operation. With the strict reproduction of all synthesis conditions, the properties of the films were also reproducible.

- During the formation of Fe_3_O_4_ particles in films, Fe(III) and Fe(II) salt concentrations of 0.10 and 0.05 mol/dm^3^, respectively, were selected. To obtain smaller and more homogeneous magnetite particles, a 2.5% ammonia solution was placed in the desiccator. To meet the conditions necessary for obtaining color-homogeneous sensor elements, in addition to using the fan, the method of impregnating the polymer with iron salts was modified. The films were kept in solutions for different periods under the influence of ultrasonic radiation to achieve a more uniform and faster penetration of iron salts into the gel. The magnetic platform in the desiccator, on which the films were placed, contributed to a more uniform distribution of growing Fe_3_O_4_ particles throughout the entire volume of the films. Table 3 shows photos in transmitted light of the films obtained at different times of impregnation with iron salts. The growth time of submicron Fe_3_O_4_ particles in all cases was 15 min. As the amount of magnetite increased, the composites took on a darker shade of brown. Starting from the third sample, the composites have almost the same color, which is probably due to the achievement of the maximum saturation of the polymer with iron salts.

The application of Formula (2) for the calculation of the analytical signal described further requires measurements of both the blank and the analyzed sample under the same conditions. However, the use of all three color coordinates instead of one makes it possible to expand the range of detectable component contents and increase the sensitivity of their determination. This approach was tested on model electrolyte solutions, in which the behavior of cross-linked PVA was previously well studied. Thus, when using all three color coordinates, instead of just one, for a film with the highest content of Fe_3_O_4_ particles, the sensitivity coefficient increases from 6.1 to 45.9 dm^3^/mol, an increase of almost 7.5 times. At the same time, the film does not lose its sensory properties for at least 5–7 analytical cycles, which is also consistent with the results obtained using the optical micrometry method. A single analytical cycle consists of moving a composite film from distilled water into an analyte solution, swelling (or compressing) the film until equilibrium is reached and then measuring the response, after which the film can be moved back into distilled water to restore its original parameters.

#### 2.2.2. Analysis of Model Solutions of Alcohols

It is known [33] that materials made of swelling polymers are suitable for the analysis of water–alcohol systems with high alcohol contents. Polyvinyl alcohol also belongs to such materials. In this work, composite films with a “crosslinked PVA—magnetite” composition were investigated as sensitive elements for determining the volume fractions of ethanol and isopropanol in aqueous alcohol solutions. The choice of analytes was due to their widespread use in everyday life and in laboratories.

**Figure 5:**
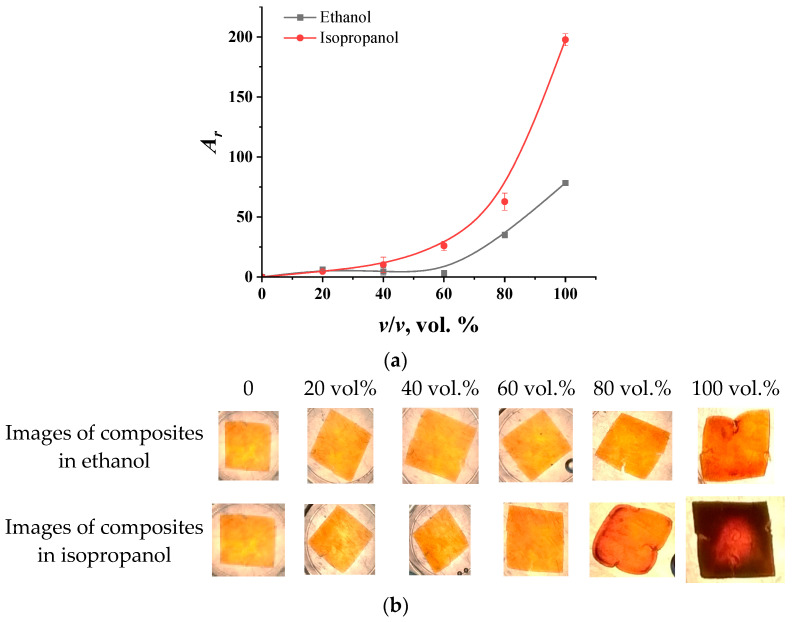
The dependence of the intensity of the *A_r_* color parameters on the volume fraction of alcohols in individual aqueous alcohol solutions (**a**) and photographs of the films after exposure in solutions (**b**).

As for electrolyte solutions, concentration dependencies of the intensity of colorimetric parameters (*A*_r_) for a film aged for 2 min in iron salts were obtained for aqueous alcohol solutions. The choice of the film was due to the highest contrast of the images of the swollen and compressed films, since, when alcohol is exposed to the PVA gel, the film is mainly compressed. The obtained graphs of the dependence of the signal on the volume fractions of alcohols are shown in Figure 5. As can be seen from the photos below, with an increase in the proportion of alcohol in the solution, the film becomes darker.

These data are in good agreement with the results obtained using optical micrometry in the study of the behavior of composite granules in aqueous alcohol solutions [34]. However, the difference lies in the sensitivity of the film to pure alcohols with almost the same volume of gel in them. This is probably due to the fact that the isopropanol, owing to its larger molecular size, is less readily sorbed by polyvinyl alcohol, since it penetrates the polymer solution phase less effectively. As a result, the unsorbed part of the isopropanol molecules is likely sorbed by magnetite due to the formation of hydrogen bonds with hydroxyl groups on the surface of the Fe_3_O_4_. This leads to the formation of “isopropanol—magnetite” aggregates, which, due to the peculiarities of the orientation of alcohol in them, begin to approach each other, which leads to a more drastic decrease in signal intensity. This assumption can also be confirmed by the fact that a noticeable response to ethanol is observed starting from a 60% solution, while the concentration dependence of the signal for isopropanol follows a smoother, monotonously increasing curve. It should be noted that when exposed to alcohols, composite films withstood at least 5–7 cycles of operation.

For using films for analytical purposes, it is convenient to use linear dependencies. For ethanol, the linear dependence of the signal on the concentration is observed in a high range of contents (in 60 vol. % and above). To obtain a linear concentration dependence with the signal for isopropanol, the value of the analytical signal was calculated in logarithmic form. Based on these linear dependencies, it was found that the detection limit of ethanol is 63 vol. %, while that of isopropanol is 24 vol. %. Thus, composite films with “PVA—magnetite” compositions are best suited for analyzing objects with high alcohol contents to determine or assess their purity.

#### 2.2.3. Determination of the Ethanol Content in Hand Antiseptics

The method of digital colorimetry using composite films composed of “crosslinked polyvinyl alcohol—magnetite” was tested for determining the volume fraction of alcohol in hand antiseptics of Dettol Original © and Sanitelle © brands based on ethanol. The results obtained (Table 4) were compared with the values declared by the manufacturers on the packages. The following composition is indicated on the packaging of Sanitelle © hand antiseptic: 66.2% ethyl alcohol, deionized water, glycerin, propylene glycol, Aloe Vera extract, vitamin E, and functional additives. The Dettol Original © package contains the composition: 68% denatured ethyl alcohol, deionized water, propylene glycol, copolymer of polyethylene glycol and polypropylene glycol, tetrahydroxypropyl ethylenediamine, acrylates, and perfume additives.

## 3. Discussion

So, the introduction of magnetite particles into polymer granules gives them color and contrast. This is an advantage when measuring the swelling of a granule in an analyte solution using optical micrometry. Moreover, it helps to fix the composite granules with a magnetic field during measurements in flow systems; as a result, the reproducibility of the analysis can be increased. For polymer films, the presence of magnetite particles is responsible not only for the color of the film, but also for its changes with an increase or decrease in the degree of swelling. In this case, the analytical response can be measured using digital colorimetry.

Currently, a limitation of the proposed approach is the relatively long time required to achieve equilibrium. The time to achieve equilibrium can be reduced by decreasing the thickness of the films used, within reasonable limits to avoid the loss of mechanical strength. It should be noted that commercial alcohol and water–alcohol solutions used in everyday life often contain additives of dyes, which can complicate the analysis. One possible solution is to subtract the background signal of the matrix from the analytical color signal received from the sample itself. An alternative approach for measuring colored media is to calibrate the photo camera according to standard templates, which can be used to find correction factors for the color of the sample. Such measurements will be the subject of further research.

The most suitable objects for analysis are antiseptics, disinfectants, and perfumes. The use of composite films is not limited to the determination of alcohols alone. Such materials, in the form of thin films, also can be used easily to determine organic acids and amino acids, provided it is possible to achieve the necessary selectivity, which depends on both the nature of the polymer and the nanoparticles. Carbohydrates can also be determined using such films as those demonstrated using optical micrometry (Section 2.1). Since films have properties similar to photonic crystals, it is possible to improve the method of colorimetry with photography in reflected light, which will equally allow all three color coordinates to be used [27,28,35,36,37]. As a prospect for the further development of the approach, it is worth noting the potential for varying the nature of the polymer matrix. This could include the use of natural polymers, such as starch and cellulose, or other synthetic matrices, such as pyridine-containing polymers, which have a higher degree of swelling than PVA [38,39]. Another promising direction of research may be a change in the nature of the nanoparticles embedded in the polymer gel. Therefore, the use of composite films based on swelling polymers in combination with digital colorimetry is a new step for the development of out-of-laboratory analysis.

## 4. Materials and Methods

The following reagents were used in this work: polyvinyl alcohol PVA 18/11 hydrolyzed, NaOH that was “pure for analysis”, epichlorohydrin (Sigma-Aldrich, Saint Louis, MO, USA), FeCl_3_·6H_2_O that was “pure”, (NH_4_)_2_Fe(SO_4_)_2_·6H_2_O that was “pure for analysis”, 25% ammonia that was “pure for analysis”, Na_2_B_4_O_7_ × 10H_2_O that was “pure for analysis”, Na_2_HPO_4_ that was “pure”, KH_2_PO_4_ that was “pure”, ethanol (95%, “Reachim”), isopropanol (pure, “Chimprolab”), and deionized water.

### 4.1. Synthesis of a Polymer Matrix Based on Cross-Linked PVA

Films and spherical granules from cross-linked PVA were obtained using a modified technique proposed in [33]. A highly alkaline solution of polyvinyl alcohol was prepared to activate OH groups as nucleophilic centers. A 20 g PVA suspension was kept in 100 cm^3^ of distilled water, then heated to a temperature of 90–100 °C until the polymer was completely dissolved and air bubbles were removed (15–30 min). The resulting solution was transferred to a 800 cm^3^ glass, immersed in a thermostat at a temperature of 70 ± 1 °C. Under continuous stirring, a solution of 10 g of NaOH was poured drop by drop into 20 cm^3^ of water for 30 min. Next, the PVA was cross-linked with epichlorohydrin in an emulsion. To obtain granules, the rotation speed of the agitator was increased to 850 rpm, 20 cm^3^ of epichlorohydrin was added, and 300 cm^3^ of I-40A engine oil was added a minute later. The system was left under continuous stirring at the same speed and the same temperature for 3 h. The obtained granules were transferred to water, and washed from oil residues sequentially with petroleum ether, isopropyl alcohol, and distilled water. The granules were stored in a jug with distilled water. To obtain films, 20 cm^3^ of epichlorohydrin was added with rapid stirring at a temperature of 30–40 °C and the resulting gel was poured into pre-prepared molds with a depth of about 1 mm and left under pressure for a day. The films from the cross-linked PVA were washed with deionized water, ethanol, and again with deionized water.

### 4.2. Formation of Magnetite Particles inside Cross-Linked PVA Gel

Composite materials of “PVA-magnetite” in the form of spherical granules or films were obtained in a desiccator with a standard fan mounted in the lid (rotation speed 2600 rpm, power 2.64 W). The PVA granules, pre-washed, were kept in a solution of a mixture of iron (III) and (II) salts in a 2:1 ratio according to the developed method [31]. They were then placed in a desiccator, at the bottom of which a Petri dish with a concentrated ammonia solution was placed. The fan was turned on so that ammonia vapors were evenly distributed throughout the entire volume of the desiccator and the granules were kept there for a day. They were then removed from the desiccator, transferred to the test tube, and repeatedly washed with distilled water. If necessary, they were mixed with distilled water in a conical flask using a shaker to remove iron oxide particles from their surface. The cleaned granules were then used for further experiments. The films of cross-linked PVA were cut into square plates measuring 0.7 × 0.7 cm, soaked in a solution of iron (III) and iron (II) salts with a 2:1 concentration ratio, and kept in an ultrasonic bath (47 kHz, 60 W). The precursors prepared in this way were placed on the magnetic platform of the desiccator, at the bottom of which there was a 2.5% NH_3_ solution, and kept there for 15 min. The resulting composite films were repeatedly washed with distilled water to remove iron and magnetite salt residues from the surface and stored in buckets filled with distilled water.

### 4.3. Preparation of Supporting Solutions

Measured amounts of 16.0 g of NaCl, 0.4 g of KCl, 7.27 g of Na_2_HPO_4_, 0.49 g of KH_2_PO_4_, and the required amount of Na_2_B_4_O_7_·10H_2_O were placed in a beaker with a capacity of 1000 cm^3^. The salts were dissolved in water when heated in a water bath, then cooled to room temperature and adjusted to the required pH value by adding concentrated HCl or NaOH solutions, controlling the acidity using a pH meter. Then, the resulting solution was quantitatively transferred to a 2000.0 mL volumetric flask, brought to the mark with distilled water, and mixed. The background solutions obtained in this way were used to prepare solutions containing glucose, fructose, or sucrose.

### 4.4. Measurements Using Optical Micrometry and Digital Colorimetry

PVA granules (5–12 pcs.), previously brought into equilibrium with water or a supporting solution, were transferred into the tablet with 48 cells for biochemical studies, filled with the same solvent/supporting solution, and covered with a cover glass. The granules were photographed using a Celectron digital video camera placed in an optical microscope equipped with a 3.7× magnification lens, and the initial volume of the granule (*V*_0_) was measured from the obtained digital photographic images. After that, water or a supporting solution was taken with a medical syringe with a soft tip with an inner diameter of 0.34 mm, and a test solution containing a carbohydrate was poured into the cells. The cells were covered with a cover glass and the granules were left until equilibrium was established (40–50 min). The granules were photographed again, and the volume of the granules that had reached equilibrium with the studied solutions was measured. For each solution, the average value of *V*/*V*_0_ and the confidence interval were calculated and the signal dependencies on the concentration or pH of the studied solution were plotted.

For the colorimetric measurements of the compositions of solutions using films, we proposed an installation that included a smartphone and a tripod equipped with a lighting source. The measured sample was placed on a slide table so that the light from the source passed through the composite film. A special smartphone mount allowed us to use smartphones of any size.

During the analysis, the composite film was photographed in a cell filled with distilled water. Then, the composite was moved to the test solution and kept for the time necessary to achieve an equilibrium degree of swelling (at least 20 min [25]). After that, the film was photographed again in a cell filled with the test solution. The photos of the composite obtained in this way in the water and the test solution were transferred to a computer and the color coordinates in an RGB space were calculated in the Wayne Rasband ImageJ © program [40]. The value of the analytical signal—the intensity of the *A*_r_ color parameters—was calculated using the following formula:

(2) $$A_{r}=\sqrt{{\left(R-R_{0}\right)}^{2}+{\left(G-G_{0}\right)}^{2}+{\left(B-B_{0}\right)}^{2}}$$

where *R*_0_, *G*_0_, *B*_0_, *R*, *G*, and *B* are the digital values of the intensities of red, green, and blue colors for the composite in water and the analyzed solution, respectively [27,28].

## 5. Conclusions

The preparation of a composite based on crosslinked polyvinyl alcohol with uniformly distributed submicron magnetite particles was described, and the possibility of its use as a stimuli-responsive material in various forms (spherical granules and thin films) for the analysis of real objects using optical micrometry and digital colorimetry was demonstrated. The limit of determination of both glucose and fructose found using optical micrometry was 7.9 mmol/dm^3^. A study of the possibility of the determination of ethanol and isopropanol demonstrated the limit of determination to be 63 and 24 vol. %. The results obtained by the use of sensing systems with composites performed with a high amount of measurement accuracy and simplicity.

The embedding of Fe_3_O_4_ particles in polymer gels is a promising area for the construction of many sensing devices and platforms. Magnetic properties of Fe_3_O_4_ have already been used for the synthesis of sorbents for the preconcentration of analytes. Additionally, magnetite particles allow for the fixing of granules in measurement cells using magnets in optical micrometry methods. Another possible route for the use of magnetic composites is the fabrication of photonic crystals utilizing the large variety of stimuli-responsible polymers, which can be a perspective area for future investigations.

## Figures and Tables

**Table 1:** The amount of magnetite in composite granules of “cross-linked PVA—magnetite”.

*C*_Fe(III)_, mol/dm^3^	*C*_Fe(II)_, mol/dm^3^	*X*_magnetite_, mass. %
0.02	0.01	0.44
0.05	0.025	1.54
0.10	0.05	3.63

**Table 2:** The amounts of magnetite in composite granules of “cross-linked PVA—magnetite”.

Sample	Declared by the Manufacturer, Mass. % (or Determined by an Independent Method—Iodometric Titration—for Honey)	Found, Mass. %	RSD, % (for Optical Micrometry)
Date syrup	77.0	78.4 ± 4.6	2.90
Agave syrup	78.0	78.0 ± 3.4	1.38
Linden honey	71.8	70.5 ± 2.0	2.85
Buckwheat honey	59.4	61.5 ± 1.1	1.82

**Table 3:** The holding times of the films in a solution of iron salts.

Holding Time, Min	1	2	3	4	5	10
Photographs of composite films in water	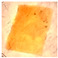	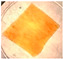	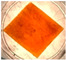	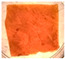	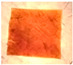	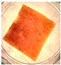

**Table 4:** The results of determining the volume proportion of ethanol (*v*/*v*) in hand antiseptics (*n* = 3, *p* = 0.95) and evaluating the accuracy of the method.

Antiseptic	Declared by the Manufacturer, vol. %	Found in the Sample (without Additives), vol. %	Added, vol. %	Found, vol. %	Relative Error, %
Dettol original (Reckitt, Great Britain; https://reckitt.com/)	68.0	66 ± 3	10	76 ± 5	3.2
Sanitelle (Bentus Laboratories LLC, Moscow, Russia; https://bentuslab.ru)	66.2	6 5 ± 3	10	75 ± 5	1.3

## Data Availability

The data presented in this study are available on request from the corresponding author.
